# User-Centered Design to Enhance mHealth Systems for Individuals With Dexterity Impairments: Accessibility and Usability Study

**DOI:** 10.2196/23794

**Published:** 2022-02-24

**Authors:** Kuntal Chowdhary, Daihua Xie Yu, Gede Pramana, Matthew Mesoros, Andrea Fairman, Brad Edward Dicianno, Bambang Parmanto

**Affiliations:** 1 Department of Physical Medicine and Rehabilitation University of Pittsburgh School of Medicine Pittsburgh, PA United States; 2 Department of Health Information Management School of Health and Rehabilitation Sciences University of Pittsburgh Pittsburgh, PA United States; 3 Human Engineering Research Laboratories Department of Veterans Affairs Veterans Affairs Pittsburgh Healthcare System Pittsburgh, PA United States; 4 Occupational Therapy Doctorate Program Johnson & Wales University Providence, RI United States

**Keywords:** cellular phone, mobile apps, telemedicine, adaptive mHealth, rehabilitation, self-care, spinal cord injury, spina bifida, chronic disease, persons with disability, accessibility, dexterity impairments, mobile phone

## Abstract

**Background:**

Mobile health systems have been shown to be useful in supporting self-management by promoting adherence to schedules and longitudinal health interventions, especially in people with disabilities. The Interactive Mobile Health and Rehabilitation (iMHere) system was developed to empower people with disabilities and those with chronic conditions with supports needed for self-management and independent living. Since the first iteration of the iMHere 1.0 app, several studies have evaluated the accessibility and usability of the system. Potential opportunities to improve and simplify the user interface were identified, and the iMHere modules were redesigned accordingly.

**Objective:**

In this study, we aim to evaluate the usability of the redesigned modules within the iMHere 1.0 app.

**Methods:**

We evaluated the original and redesigned iMHere modules—MyMeds and SkinCare. The Purdue Pegboard Test was administered to assess the participants’ dexterity levels. Participants were then asked to perform a set of tasks using both the original and redesigned MyMeds and SkinCare modules to assess their efficiency and effectiveness. Usability was measured using the Telehealth Usability Questionnaire to evaluate 10 new accessibility features that were added to the redesigned app. Participants were also asked which version they preferred.

**Results:**

In total, 24 participants with disabilities and varying degrees of dexterity impairments completed the entire study protocol. Participants displayed improved efficiency and effectiveness when using the redesigned modules compared with the original modules. The participants also reported improved usability and preferred the redesigned modules.

**Conclusions:**

This study demonstrated that the iMHere system became more efficient, effective, and usable for individuals with dexterity impairments after redesigning it according to user-centered principles.

## Introduction

### Background

The advent of smartphones has transcended the mobile phone’s original purpose—the ability to make phone calls anywhere. Notably, smartphones have radically altered the way people communicate with friends and family, coordinate daily activities, and organize their lives. At the most fundamental level, smartphone users expect their devices to provide an immediate and reliable means of managing their everyday lives [1,2].

One of the most significant emerging trends in the health-related use of smartphones is the proliferation of mobile health (mHealth) apps. These apps can be implemented in a variety of settings, with many focusing on monitoring, managing, and supporting health-related behavior changes [3]. One of the most common type of health-related app focuses on the management of chronic conditions, such as obesity, chronic pain, and type 2 diabetes mellitus, through patient empowerment [4-6].

People with disabilities, however, are one of the largest populations facing health issues that limit their function and participation. The World Health Organization estimates that over 1 billion people, about 15% of the world’s population, live with some form of disability [7]. As the population continues to age, the rate of disability continues to rise, in part owing to chronic conditions and the effects of aging itself. Many people with disabilities also have limited access to health care.

Given the high degree of portability and adaptability, mHealth can facilitate self-management and community integration by providing support when the user is between medical visits or in any location, including outside the home. These features may be particularly useful in supporting people with disabilities, who often have limited access to health care and community-based resources to support independent living. The support provided by mHealth may allow users to address secondary complications, which are not always addressed adequately in the outpatient setting, thereby reducing the cost of care [8-10]. Strong evidence supports the importance of using tools to promote self-management skills to improve the health outcomes and independence of people with disabilities [11,12].

Despite the need for mHealth tools to support self-management, a Pew Research Center survey in 2016 found that 65% of people with disabilities have low confidence in their ability to use the internet and other communication devices to keep up with information [13]. This is further compounded by a general lack of awareness of the accessibility features of apps and the skills to use mobile devices optimally [14]. In addition, many mainstream mHealth apps are not designed to address usability or accessibility [15].

**Figure 1:**
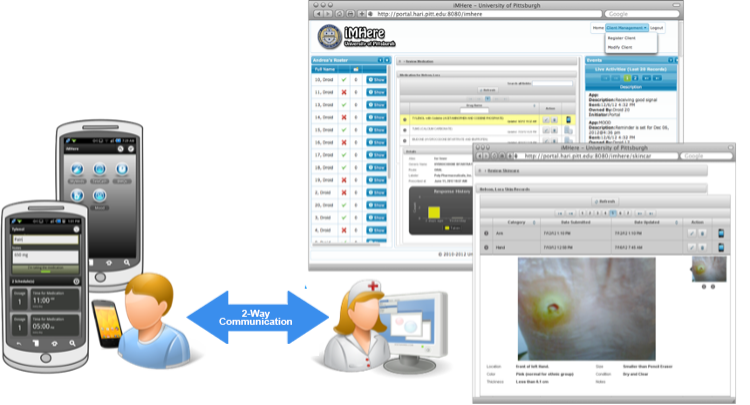
Interactive Mobile Health and Rehabilitation platform—MyMeds and SkinCare modules as seen by user.

The Interactive Mobile Health and Rehabilitation (iMHere) system is an mHealth system that was developed to empower people with disabilities and those with chronic conditions with the skills needed for self-management and independent living [16]. The iMHere 1.0 system originally consisted of a smartphone app for people with disabilities and a web-based portal for clinicians, bridged by a 2-way communication protocol (Figure 1).

The iMHere 1.0 app comprised a suite of 5 modules to support medication management (MyMeds), skin integrity (SkinCare), bowel management, bladder self-catheterization, and mental health. People with disabilities could use this suite of modules to report compliance with treatment regimens, ask questions, and receive personalized treatment plans, educational materials, and messages from the clinician. On the clinician side, a web-based monitoring portal allowed clinicians to engage with patients and track their adherence to a specific and individualized treatment plan. By accessing the iMHere portal, clinicians were able to monitor patients’ adherence to self-management activities, view reported problems and issues, and send personalized treatment plans to patients [16].

Given the vast health care implications of using mHealth solutions in people with disabilities, usability testing of mHealth apps is needed. Usability testing has been widely used in the people with disabilities population to test mobile self-management programs. Payne et al [17] demonstrated that usability testing of a web-based e-counseling platform to promote behavioral self-management in patients with chronic heart failure had favorable outcomes in improving the navigation of the website. Williams et al [18] also assessed the usability of a pediatric cardiovascular disease risk factor tool, which yielded revisions through tester feedback to make the mobile app more user-friendly. Thirumalai et al [19] evaluated the development process of a telehealth app used by people with multiple sclerosis through a usability study, which incorporated revisions into the final app. These previous works highlight the importance of usability testing, as it can help identify issues specific to the people with disabilities population that may not have been addressed by program developers in the first iteration of the mHealth solution.

We conducted extensive user acceptance and usability testing of the iMHere system. In the past, 3 studies on the accessibility of iMHere 1.0 have been conducted. In the first study by Yu et al [20], the iMHere 1.0 system was tested for usability. In this study, the modules were tested for self-management workflow, user interface and navigation, and patient-clinician communication. All participants in the study were interested in daily use of the phone app, with the MyMeds and SkinCare modules used frequently by all users, as demonstrated by the consistent use of the phone app during the 6-month intervention period. The clinical portal allowed clinicians to continually monitor patients’ conditions and take appropriate steps to prevent secondary complications [20].

In a subsequent study by Yu et al [21], the iMHere 1.0 app was tested for accessibility in 6 participants with spina bifida (SB). The study specifically explored participant experiences with the user interface and the navigation of the module. All 6 participants viewed the modules positively with regard to their support for self-management activities, as indicated by the Telehealth Usability Questionnaire (TUQ) scores (6.52/7 points, 93%). This was further strengthened by the efficiency of performance, as it was noted that shorter times to complete tasks and reduced error rates were seen over repeated trials. In this study, a few avenues for improvement to accessibility were identified, including the need for changes to accommodate users with dexterity impairments.

In a subsequent study, Yu et al [22] explored the accessibility needs and preferences of iMHere users with various disabilities that lead to dexterity impairments. Participants completed 5 tasks, and the *difficulty-on-performance* (DP) was calculated. As expected, a higher degree of dexterity impairment demonstrated more problems in task completion. A few potential issues and barriers were identified, including changes needed to the user interface to create a consistent design, instructive guidance, and simpler cognitive processes in the use of the app.

### Objectives

The modules within iMHere 1.0 were redesigned based on these prior studies. The aim of this study is to evaluate the usability of redesigned modules within iMHere 1.0. Hypothesis 1 was that usability (as defined by efficiency and effectiveness) would be higher when completing tasks in the redesigned modules compared with the original modules. Hypothesis 2 was that usability (as measured by the TUQ, which evaluates learnability and satisfaction) would be higher in the redesigned modules, compared with the original modules [23].

## Methods

### Overview

#### Modules

This study was designed to evaluate the usability of two modules of the iMHere system: the original and redesigned versions of MyMeds and SkinCare. These modules were specifically selected because of the high rates of medication use and pressure ulcers in the people with disabilities population. Medication mismanagement and inadequate care of pressure injuries are the causes of high rates of hospitalization in the people with disabilities population and significantly increase morbidity [24-26]. These modules were also the most complex iMHere modules in terms of functionality.

#### MyMeds Module

The MyMeds module helps users manage their medications by providing reminders and monitoring medication adherence. Persons with conditions, such as SB and spinal cord injuries (SCIs), for example, are frequently prescribed several medications for managing neurogenic bowel, neurogenic bladder, spasticity, pain, and depression. Taking multiple medications multiple times per day, while at the same time having to consistently follow other complex self-management routines can be particularly challenging. The MyMeds module helps patients adhere to their medication regimens by providing reminders or cues, keeping track of all their medications and medication schedules (including those medications currently prescribed or prescribed in the past), and reporting if and when the medications have been taken.

#### SkinCare Module

The SkinCare module reminds users to perform routine inspections of their skin, enables users to take pictures and track any wound or skin conditions that have developed, and at the same time provides the ability to communicate with clinicians on how to care for these problems. For people with SB or SCI; for example, constant vigilance is needed to prevent pressure injuries, particularly in the lower limbs and buttocks, where sensation may be impaired. Pressure injuries are not only detrimental to the patient owing to increased mortality and increased intensive care unit and hospital length of stay but also present a significant health care burden given increased health care costs and health care use following discharge [27-29].

### Study Design

This study was approved by the Institutional Review Board of the University of Pittsburgh (PRO12090453). All participants provided informed consent for participation. The participants were recruited from local outpatient rehabilitation medicine clinics. A sample size calculation was performed using the Wilcoxon signed-rank test (2-sided). A sample size of 14 achieved 91% power to detect a mean of paired differences of 1.0, with an estimated SD of paired differences of 1.0, with a significance level (α) of .05.

The inclusion criteria were as follows: users must be between the ages of 18 and 64 years, have fine motor dexterity impairments, have potential for skin breakdown (defined by diagnosis or lack of sensation), and use at least one (prescription or nonprescription) medication. The exclusion criteria were as follows: users with vision, hearing, or speech limitations that entirely precluded the use of a smartphone. Individuals were not excluded if they had used iMHere in a prior study, but a 4-month washout period was used to mitigate learning effects.

Usability was defined according to the usability attributes by Nielsen [30]. The Nielsen model of usability was selected as a framework for this study, as it is multifaceted in its approach to the many dimensions of usability. We examined the usability constructs of efficiency and effectiveness (including errors; hypothesis 1) by assessing task time and errors made. We also used a validated usability survey (TUQ) to measure the usability constructs of learnability and satisfaction (hypothesis 2). We also evaluated user preferences. This design has been used in prior research [31-34]. Participants were first randomly assigned to use either the original or redesigned modules. Participants were then crossed over and provided with alternate modules, such that each participant served as his or her own matched control. As such, we elected not to test memorability in this study, as testing of memorability would confound our washout period between testing of the original and redesigned modules. Data were collected either in the laboratory or at the site of the participant's choosing (ie, home or office).

### Demographics, Training, and Dexterity

A background questionnaire was administered to collect the participants’ demographic data, previous experience with mobile phones, and knowledge of mHealth technologies.

A face-to-face orientation and training session (approximately 15 minutes) was conducted to introduce the MyMeds and SkinCare modules. Participants were trained to perform the tasks for each of the modules using a trial medication bottle and a mock skin problem image. Participants were scheduled to complete the second set of modules after a 3-week period. This crossover period served as the washout period to minimize the learning effects.

To assess the participants’ dexterity levels, the Purdue Pegboard Test (PPBT) was administered to measure the movements of a person’s fingers, hands, and arms [35-39]. The PPBT was initially developed by Joseph Tiffin in 1948 to test the manual dexterity of those seeking employment in industrial jobs, such as factory workers on an assembly line. Although most individuals no longer have occupations akin to factory workers, technological advancements have created new requirements for high dexterity, such as typing on a computer keyboard or messaging on a cell phone. Despite the cultural shifts in the past few decades to include technology such as mobile devices, the PPBT has been shown to be valid and reliable for wrist and hand disorders and has since been adapted for use in testing dexterity in the clinical setting [40,41].

The PPBT consists of 3 tests at 30-second intervals using the right hand, left hand, and both hands. In each test, participants were asked to pick up pins, collars, or washers from the top of the pegboard and drop them in the peg holes as rapidly as possible in 30 seconds. The score for each test was based on the total number of pins, collars, or washers that dropped in the holes correctly. A composite score was calculated by summing the scores from these 3 tests, yielding the *right+left+both hands* score. This score represents participants’ overall dexterity levels. Lower *right+left+both hands* scores indicate a higher degree of dexterity impairment. On the basis of their *right+left+both hands* scores, participants were categorized into the following 3 groups reflecting their dexterity levels:

- Group 1: users with mild dexterity issues as defined by PPBT scores for the *right+left+both hands* scores ranging from 1 SD to 3 SD below the generic mean of factory workers.
- Group 2: users with moderate dexterity issues as defined by *right+left+both hands* scores >3 SD below the generic mean of factory workers.
- Group 3: users with severe dexterity issues as defined by the inability to perform the PPBT (*right+left+both hands* score=0).

### Efficiency and Effectiveness

Participants were then asked to perform a set of tasks using both the original and redesigned MyMeds and SkinCare modules. The *think aloud* method for product design and development was used to gain comprehensive knowledge of participants’ experiences, including any experienced frustration [42]. Specifically, participants were asked to verbally describe their intentions and actions to the researcher as they performed the following tasks:

- Task 1: schedule a new medication—participants were asked to locate the correct medication, add information about their regimen, and set up a reminder.
- Task 2: modify a medication reminder—participants were asked to change the alert time for medication.
- Task 3: respond to a medication alert—participants were asked to indicate whether a medication was taken.
- Task 4: set up a schedule to check the skin—participants were asked to set a daily alert to conduct a skin evaluation.
- Task 5: modify an alert for skin check—participants were asked to change the alert time for the scheduled skin evaluation.
- Task 6: report a skin issue—participants were asked to identify a skin issue, and then take a picture and enter data into predefined fields within the module, describing the affected skin region, including location, color, size, depth, and tissue condition.
- Task 7: update or track changes in an existing skin issue—participants were asked to reassess previously identified skin issues and track changes by taking pictures and filling out a form describing the affected skin region, including location, color, size, depth, and tissue condition.
- Task 8: set personalized configurations for user interface presentations—participants were asked to record a preferred module list, background, text size, and target size to optimize interactions. This task was conducted only for the redesigned module.

Task 8 was performed only when a participant was testing the redesigned modules.

The following variables were collected:

- Efficiency
  Average task time: the time in seconds for a participant to complete each task was measured and then averaged across all 8 tasks.
- Effectiveness
  Number of steps in each task: number of actions taken by the participant to complete a given task.
  Number of mistakes in each task: when a participant reported a problem finishing a task, it was counted as a mistake. Mistakes were tallied to each task.
  Error rate: the sum of mistakes divided by the total number of steps required to complete a task.
  Mistake recovery: ability of participants to correct mistakes. Step-by-step observation notes were used to record the status of mistake recoveries, which were used to describe the DP experienced by a participant during mistake recovery. The DP score was calculated as the sum of weighted scores, where a lower DP score indicated better and easier performance on the task.
    1. The participant solved the problem without any help.
    2. The participant needed help solving the problem, addressed in one sentence.
    3. The participant needed help solving the problem, addressed in 2-4 sentences.
    4. The participant did not solve the problem.

### Learnability and Satisfaction

#### Overview

Usability was measured using TUQ (Table 1). The TUQ measures constructs of usability, such as learnability and satisfaction. Learnability, as defined by Nielsen [30], assesses how easily users can accomplish a task the first time they encounter the interface and how many repetitions it takes for them to become efficient at that task. The TUQ has been shown to have high validity, reliability, and internal consistency [23]. It provides a more comprehensive evaluation of telehealth tools, given that it has combined existing sources in telemedicine (such as the Telemedicine Satisfaction Questionnaire) and computer software interface (such as the Technology Acceptance Model and the IBM Post Study System Usability Questionnaire). Participants were asked to rate the extent to which they agreed with 21 statements using a scale from 1 to 7 (minimum score 21; maximum score 147). Statements are grouped into six domains: usefulness, ease of use and learnability, interface quality, interaction quality, reliability, and satisfaction and future use. The average TUQ scores were calculated for each of the 6 domains and overall. A higher score reflects higher usability.

**Table 1:** Telehealth Usability Questionnaire items.

Components		Questionnaire items
**Usefulness**		
	1	Telehealth improves my access to health care services
	2	Telehealth saves me time traveling to a hospital or specialist clinic
	3	Telehealth provides for my health care needs
**Ease of use and learnability**		
	1	It was simple to use this system
	2	It was easy to learn to use the system
	3	I believe I could become productive quickly using this system
**Interface quality**		
	1	The way I interact with this system is pleasant
	2	I like using the system
	3	The system is simple and easy to understand
	4	This system is able to do everything I would want it to be able to do
**Interaction quality**		
	1	I could easily talk to the clinician using the telehealth system
	2	I could hear the clinician clearly using the telehealth system
	3	I felt I was able to express myself effectively
	4	Using the telehealth system, I could see the clinician as well as if we met in person
**Reliability**		
	1	I think the visits provided over the telehealth system are the same as in-person visits
	2	Whenever I made a mistake using the system, I could recover easily and quickly
	3	The system gave error messages that clearly told me how to fix problems
**Satisfaction and future use**		
	1	I feel comfortable communicating with the clinician using the telehealth system
	2	Telehealth is an acceptable way to receive health care services
	3	I would use telehealth services again
	4	Overall, I am satisfied with this telehealth system

#### User Preferences

We measured user preferences by asking each participant whether they preferred the original or redesigned modules and the reasons for those preferences.

#### Accessibility

The following 10 accessibility features were demonstrated to participants in the redesigned app as part of the training during the study:

1. Customized module list: this feature provides the user with the ability to customize their app by hiding or showing a selected module from the home screen. The participants were able to personalize their home screens with the modules that were most applicable to them.
2. Customized text display: this feature allows the user to set up a reading size that is comfortable for them in the redesigned modules. The size, color, bold, and italic versions of titles, text, attention text, and warning text were predefined in the iMHere modules relative to the settings of the display text.
3. Customized theme: this feature allows the user to select their preferred background and text color.
4. Customized button size: customized button size was created after a user pressed their index finger on the screen to record her or her fingertip size. The smartphone then adapts button or icon size accordingly for all iMHere modules. Given the dexterity impairment in the study population, this feature improved the accuracy in making selections using a customized button target size.
5. Customized keyboard: the iMHere app provided a customized keyboard with softer keys, larger key sizes, and preconfigured characters. Customized keyboards were used primarily for the MyMeds module, where users could easily enter medication dosage information. When using the customized keypad to enter *2 tablets*, for instance, only 2 touches were needed, *2* and *tablet*. This customized keypad was designed to reduce the number of touches required on the smartphone screen.
6. Ability to take pictures of a pill or bottle: using this feature, users could take a photo of a pill or medication bottle and upload it into his or her medication schedule.
7. Color-coding: this feature matched the color with the module name. For instance, the title for the SkinCare module on the home page was highlighted in red. When navigating through the SkinCare modules, all screens under the module had a red bar.
8. Navigational short cut: this feature allowed users to create personalized settings for the home screen, such as a list of modules.
9. Text guidance: the modules provided short text cues with self-training instructional notes on the screen and were highlighted in a particular color.
10. Voice guidance: the modules used text-to-speech technology, which allowed users to listen to text guidance as audio output.

We asked participants to rank the importance of each accessibility feature, using a 10-point Likert scale (1 indicated that this feature was the most important and 10 indicated that this feature was the least important). The average ranking was then calculated for each accessibility feature.

### Statistical Analysis

Descriptive statistics were calculated for the demographic and usability variables, including PPBT scores.

The α level was set at .05. All statistical analyses regarding hypotheses 1 and 2 were carried out using Wilcoxon signed-rank tests. To test the first hypothesis, the original and redesigned modules were compared in terms of efficiency (average task time) and effectiveness (number of steps, number of mistakes, error rate, and mistake recovery). As some experienced users were recruited, a secondary analysis using the Mann-Whitney *U* test was used to explore whether differences in average task time for the original and redesigned modules between experienced and inexperienced users could be because of a learning effect not mitigated by the washout period. To test the second hypothesis, the original and redesigned modules were compared in terms of usability (average overall TUQ and average TUQ domain scores).

## Results

### Overview

A total of 28 participants were recruited for this study; 2 (7%) participants were excluded based on the exclusion criteria: 1 (4%) user was blind, and 1 (4%) user had both vision and dexterity impairments that precluded the use of a smartphone. Moreover, 4% (1/28) of participants was not able to complete the entire protocol because of severe dexterity impairments, as assessed by PPBT scores. In addition, 4% (1/28) of participants dropped out because of scheduling conflicts. Therefore, in total, 24 participants (n=8, 33% females and n=16, 67% males) completed the entire study protocol.

### Demographics and Dexterity

The demographics of the participants are presented in Table 2. Participants’ ages ranged from 18 to 64 years, with an average age of 28 years (SD 6.3 years). Of the 24 participants, 14 (58%) patients had SB, 5 (21%) had SCI, 3 (13%) had cerebral palsy, 1 (4%) had muscular dystrophy, and 1 (4%) had cerebellar ataxia. In total, of the 24 participants, 22 (92%) patients were right-hand dominant, 21 (88%) were smartphone users, 2 (8%) were regular mobile phone users, and 1 (4%) participant did not use any mobile device; 12 (50%) participants had used a mobile phone for <2 years, and 20 (83%) participants used a smartphone for at least 60 minutes per day. In addition, 21% (5/24) of participants had finished graduate-level education, while 71% (17/24) of participants had received a high school or equivalent education.

**Table 2:** Participant demographics (N=24).

Demographic details			Values
Age (years), mean (SD)			28 (6.3)
**Gender, n (%)**			
	Male	15 (63)	
	Female	9 (38)	
**Highest level of education, n (%)**			
	High school	17 (71)	
	Higher education	5 (21)	
**Disability, n (%)**			
	Spina bifida	14 (58)	
	Spinal cord injury	5 (21)	
	Cerebral palsy	3 (13)	
	Muscular dystrophy	1 (4)	
	Cerebellar ataxia	1 (4)	
**Type of phone, n (%)**			
	Regular	2 (8)	
	Smart	21 (88)	
	N/A^a^	1 (4)	
**Years of use, n (%)**			
	<2	12 (50)	
	>2	11 (46)	
	N/A^a^	1 (4)	
**Daily use, n (%)**			
	<60 min/day	3 (13)	
	>60 min/day	20 (83)	
	N/A^a^	1 (4)	

^a^N/A: not applicable.

Of the 24 participants, 7 (29%) participants had previously used the iMHere modules (*experienced*), and 17 (71%) participants had not previously used any iMHere modules (*inexperienced*). The experienced participants had stopped using iMHere for at least 4 months before participating in this study, a washout period that we expected the participants did not carryover significant learning from previous experience. Of the 7 experienced users, 4 (57%) participants remembered approximately 5% of the process to complete the tasks in the original modules and approximately 10% of the process in the redesigned modules. Furthermore, 43% (3/7) of participants had no recollection of how to use the modules.

All participants’ PPBT scores (*right+left+both hands*) were at least 1 SD below the generic mean (46.8, SD 4) of factory workers (Multimedia Appendix 1). There were 8 participants in group 1, 12 participants in group 2, and 5 participants in group 3.

### Efficiency: Average Task Time

Table 3 shows the average time in seconds for all participants to complete tasks 1-7 using the original and redesigned modules. The average time for the 24 participants to complete tasks 1-7 in the original modules was approximately 48 seconds. This time dropped by 35% to 31 seconds when completing the tasks using the redesigned modules. Participants’ speed in completing tasks 1, 2, 4, and 6 improved by >30% when comparing the redesigned modules with the original modules. A significant difference was found in the average task time for all tasks, except task 3, when comparing the original with the redesigned modules. Overall, a Wilcoxon signed-rank test showed that the total average task time for each participant was significantly different between the original and the redesigned modules (*W*=0.0; *Z*=−4.3; *P*<.001).

**Table 3:** Comparison of the average task time for all participants.

Tasks	Original modules (task time in seconds), mean (SD)	Redesigned modules (task time in seconds), mean (SD)	Time difference, seconds (%)	Statistics		
				*W* value	*Z* value	*P* value
Task 1: schedule a medication alert	110.5 (36.5)	68.9 (23.1)	−41.7 (−37.7)	3	−4.2	<.001
Task 2: modify a medication alert	39.6 (15.2)	25.1 (11.1)	−14.5 (−36.5)	24	−3.6	<.001
Task 3: respond to a medication alert	4.2 (3.1)	4.3 (2.9)	0.1 (1.8)	144	−0.2	.85
Task 4: schedule skin check	25.3 (11.2)	16.7 (6.6)	−8.5 (−33.7)	17	−3.8	<.001
Task 5: modify a skincare alert	21.8 (9.4)	16.5 (9.5)	−5.3 (−24.4)	56	–2.7	.007
Task 6: report a new skin problem	81.2 (17.8)	48.5 (12.0)	−32.7 (−40.2)	1	−4.3	<.001
Task 7: track the changes of a skin issue	56.0 (15.2)	38.8 (11.0)	−17.2 (−30.6)	9	−4.0	<.001

The average time in seconds to complete tasks using the original and the redesigned modules for the 29% (7/24) experienced participants and the 71% (17/24) inexperienced participants is shown in Table 4. A secondary analysis revealed no significant difference in average task time between the experienced (n=7; mean 49.0, SD 36.6) and inexperienced participants (n=17; mean 48.0, SD 37.4) when using the original modules (*U*=59; *Z*=−0.03; *P*=.98), or between the experienced (n=7; mean 31.6, SD 23.8) and inexperienced participants (n=17; mean 31.1, SD 21.7) when using the redesigned modules (*U*=59, *Z*=−0.03; *P*=.98).

**Table 4:** Experienced versus inexperienced: average task time for all participants.

Tasks	Original modules		Redesigned modules	
	Experienced (task time in seconds), mean (SD)	Inexperienced (task time in seconds), mean (SD)	Experienced (task time in seconds), mean (SD)	Inexperienced (task time in seconds), mean (SD)
Task 1: schedule a medication alert	109.0 (49.2)	111.2 (31.8)	74.1 (36.5)	66.7 (15.8)
Task 2: modify a medication alert	46.0 (19.5)	37.0 (12.9)	21.4 (7.7)	26.7 (12.1)
Task 3: respond to a medication alert	4.2 (1.6)	4.2 (3.6)	3.9 (1.2)	4.5 (3.3)
Task 4: schedule a skin check	25.4 (15.8)	25.2 (9.4)	16.5 (7.1)	16.8 (6.6)
Task 5: modify a skincare alert	21.2 (10.1)	22.0 (9.5)	18.4 (13.1)	15.7 (8.0)
Task 6: report a new skin problem	81.1 (18.4)	81.2 (18.1)	47.6 (12.7)	48.9 (12.0)
Task 7: track the changes in skin issues	58.7 (18.4)	54.9 (17.1)	39.2 (9.2)	38.7 (12.0)

As shown in Table 5, participants with severe dexterity issues (group 3) required approximately 55 seconds on average to complete the tasks using the original modules. The time to complete the tasks improved by 40% (33 seconds) using the redesigned modules, which was the largest improvement among the 3 groups. The speed of participants with mild and moderate dexterity impairments (groups 1 and 2) to complete these tasks with the redesigned modules improved by >30%.

**Table 5:** Group comparison of the average task time.

Tasks	Original modules (task time in seconds), mean (SD)	Redesigned modules (task time in seconds), mean (SD)	Time difference, seconds (%)
Group 1	44.6 (8.0)	31.7 (5.7)	−12.8 (−28.8)
Group 2	47.9 (11.4)	30.2 (6.5)	−17.7 (−37)
Group 3	54.9 (14.1)	35.8 (10.8)	−19.1 (−34.8)

The activities in task 8 for configuring personalized settings include choosing preferred modules, changing the background and text color, changing the text display size, and choosing the button or target size. Participants required approximately 36 seconds (SD 9.0 seconds) to complete this task. Specifically, participants with mild dexterity issues (group 1) spent 32.8 seconds (SD 7.07 seconds), participants with moderate dexterity issues (group 2) spent 34.4 seconds (SD 9.98 seconds), and those with severe dexterity issues (group 3) spent 42.2 seconds (SD 6.67 seconds) to complete this task.

### Effectiveness

#### Overview

Table 6 shows the total number of steps to complete the tasks, the total number of mistakes committed, the calculated error rate, and the total DP scores recorded for participants completing tasks 1-7 using the original and redesigned modules.

**Table 6:** Comparison of total steps, mistakes, and error rate.

Tasks	Original modules					Redesigned modules			
	Total steps, n	Total mistakes, n	Error rate, %	Total DP^a^	Total steps, n		Total mistakes, n	Error rate, %	Total DP
Task 1: schedule a new medication	360	32	9.3	69	264		4	1.5	8
Task 2: modify a medication alert	192	21	10.9	41	144		2	1.4	4
Task 3: respond to a medication alert	24	0	0	0	24		0	0	0
Task 4: schedule a skin check	144	5	2.9	9	120		0	0	0
Task 5: modify skin check alert	168	6	3.1	12	120		3	2.5	5
Task 6: report new skin problem	480	13	2.6	21	312		4	1.3	8
Task 7: update the existing skin problem	264	16	5.9	36	192		3	1.6	5
Total	1632	93	5.7	188	1176		16	1.4	30

^a^DP: difficulty-on-performance.

#### Number of Steps

**Figure 2:**
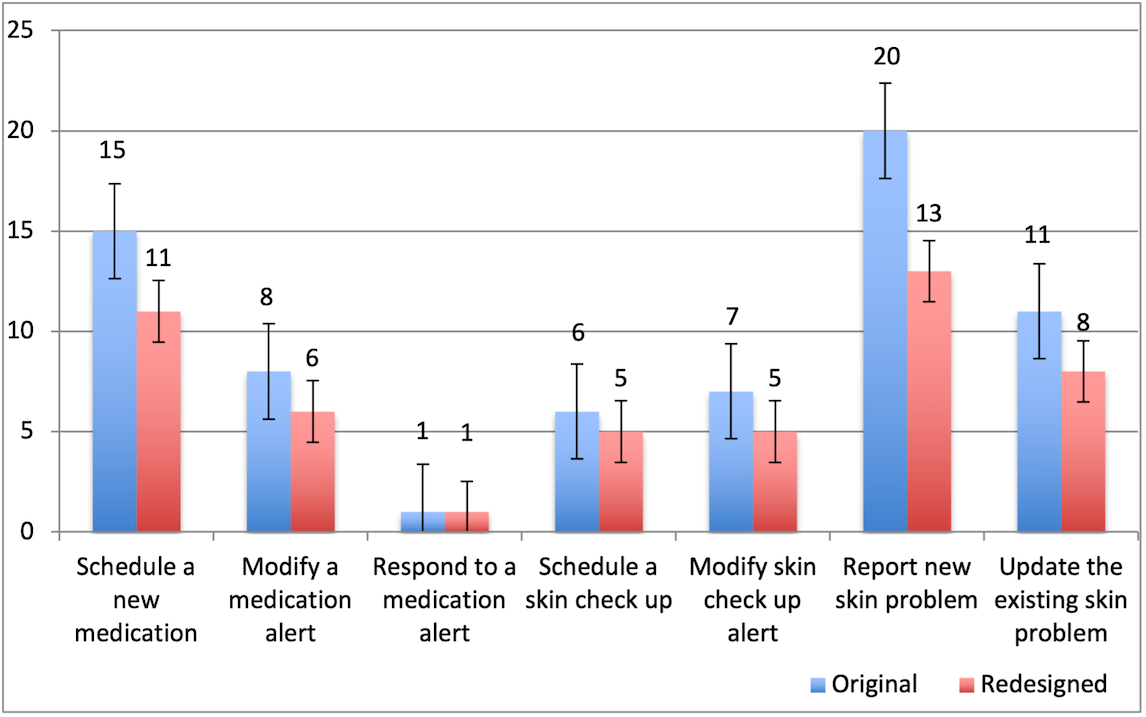
Number of steps for participants to complete tasks.

Figure 2 shows the average number of steps required by each participant to complete tasks 1-7 when using both the original and redesigned modules. On average, 68 steps (15+8+1+6+7+20+11) were required for a participant to complete tasks 1-7 using the original modules. This number dropped by approximately 25% to 49 steps (11+6+1+5+5+13+8) for the redesigned modules. In both modules, tasks 1 and 6 required the greatest number of steps to complete the task. A statistically significant difference was found in the number of steps for a participant to complete tasks in the original (mean 9.71, SD 6.26), and redesigned modules (*W*=0.0; *Z*=−2.2; *P*=.03).

#### Number of Mistakes and Error Rate

A total of 93 mistakes were identified when the participants completed the tasks using the original modules. Only 16 mistakes were identified when participants completed tasks using the redesigned modules, with an 82.8% drop rate. The reduction in the total number of mistakes for participants completing tasks 1-7 using the redesigned modules (mean 0.63, SD 1.13) compared with the original modules (mean 3.88, SD 2.66) was significantly lower (*W*=0.0; *Z*=−2.2; *P*=.03).

#### Mistake Recovery

The total DP score for participants to complete tasks 1-7 using the redesigned modules (mean 4.29, SD 3.30) was significantly lower than that for the original modules (mean 26.86, SD 23.65; *W*=0.0; *Z*=−2.2; *P*=.03).

While using the original module, participants were able to self-correct 22% (21/93) of the mistakes identified without any assistance (DP=1), 55% (52/93) after 1 sentence of assistance (DP=2), and 21% (20/93) after 2 sentences of assistance (DP=3). With the redesigned module, participants were able to self-correct 18% (3/16) of the mistakes without any assistance (DP=1), 73% (11/16) of the mistakes after 1 sentence of assistance (DP=2), and 6% (1/16) of the mistakes after 2 sentences of assistance (DP=3).

### Learnability and Satisfaction

**Figure 3:**
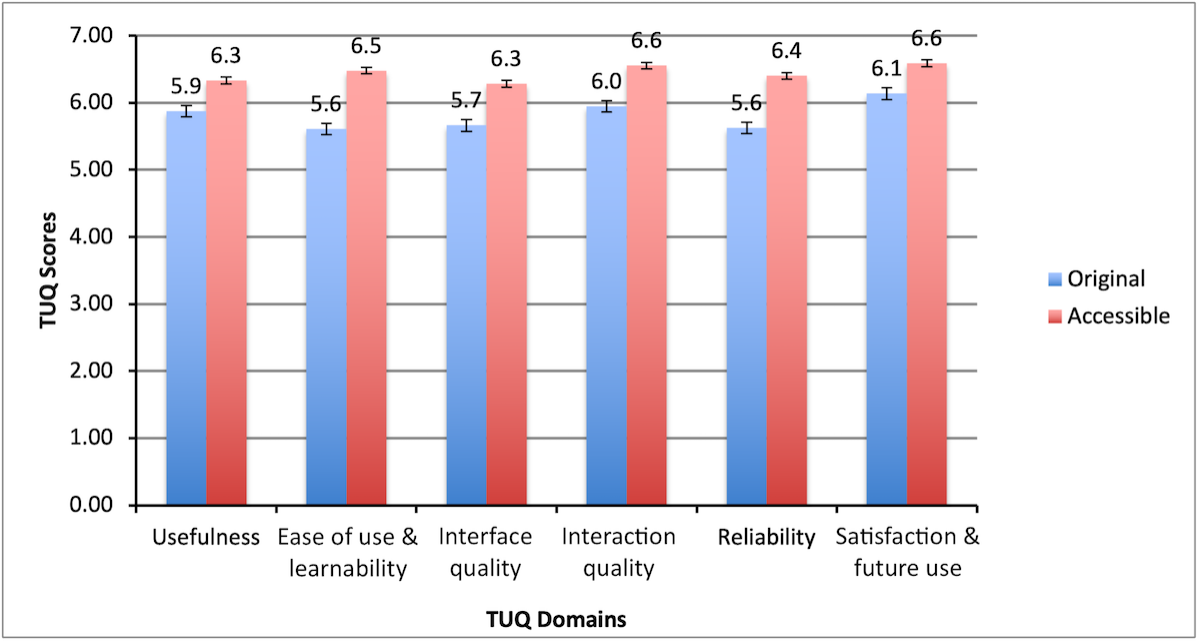
Comparison of Telehealth Usability Questionnaire (TUQ) factors and scores.

Figure 3 shows a comparison of the mean TUQ scores from the domain of the TUQ for the original and redesigned modules. On average, participants’ usability scores improved from 83% (5.86/7, SD 0.97 points) for the original modules to 92% (6.46/7 points, SD 0.53 points) for the redesigned modules, an 8.6% improvement rate. The greatest improvements in user satisfaction were noted for *ease of use and learning* (15.45%), *interface quality* (10.97%), *interaction* (10.24%), and *reliability* (13.78%). The average TUQ scores for *usefulness*, and *satisfaction and future use* increased by >7%. The difference in usability between the original and redesigned modules was significant (*W*=210; *Z*=3.9; *P*<.001).

**Figure 4:**
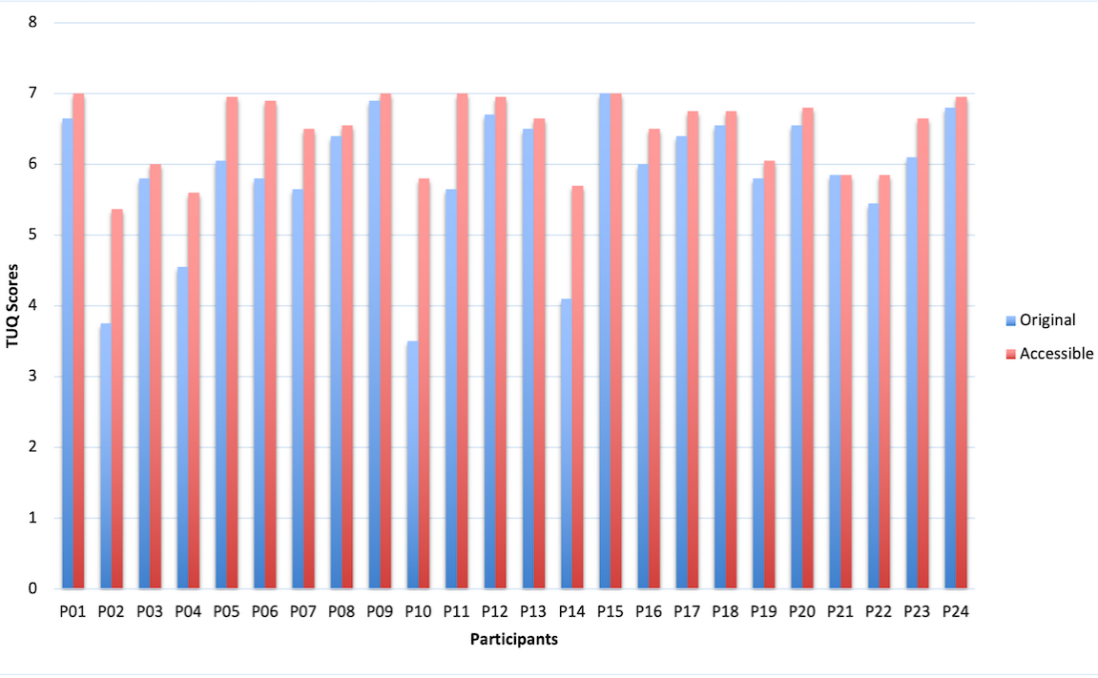
Telehealth Usability Questionnaire (TUQ) scores from participants.

Figure 4 illustrates the average overall TUQ scores for each of the 24 participants using the original and redesigned modules. With the exception of participants 15 and 21, who had the same average overall TUQ score for both modules, all other participants had higher scores for the redesigned modules. The average overall TUQ scores were significantly different when comparing scores for the original and redesigned modules (*P*<.001).

### User Preferences

Of the 24 participants, 11 (46%) tested the original modules first, followed by a test of the redesigned modules. A total of 50% (12/24) of participants tested the redesigned modules first, followed by a test of the original modules. When we asked participants’ preferences regarding the use of the original or redesigned modules, 79% (19/24) of participants indicated that they preferred the redesigned modules, 13% (3/24) possibly preferred the redesigned modules, and 4% (1/24) preferred the original modules.

Participants who preferred the redesigned modules appreciated the ease of navigation and display of the redesigned modules owing to *less typing* and *larger target*. Others found the voice guidance to be useful, stating the guide *gets user’s attention for directional notes*.

Only 4% (1/24) of participants preferred using the original modules, stating that it *looks clean* compared with the redesigned modules. This participant chose the picture of bamboo as a background in the redesigned modules, which made the redesigned modules look *busy*. However, the participant preferred the redesigned module in terms of flow in use compared with the original modules.

### Importance of Accessibility Features

Table 7 shows rankings of the 10 new accessibility features.

**Table 7:** Importance of accessibility features.

Serial no	10-item Likert scale (1=most important; 10=not important)	Average scores	Ranking based on the average scores
1	Customized module list	2.8	2
2	Customized text display	4.0	9
3	Customized theme	5.3	10
4	Customized button size	3.1	3
5	Customized keyboard	3.3	4
6	Ability to take a picture of a pill or a bottle	3.8	8
7	Color-coding	3.8	7
8	Navigational short cuts	3.5	6
9	Text guidance	2.7	1
10	Voice guidance	3.3	4

Table 8 summarizes the accessibility importance rankings grouped by dexterity levels. Regardless of their dexterity level, all participants preferred using text guidance, ranking it highly across groups. Participants with mild to moderate dexterity impairments preferred to use both voice guidance and text guidance equally. However, users with severe dexterity impairments ranked the voice guidance feature as less important. Owing to their physical limitations with respect to holding a smartphone and accessing the volume control button, participants with severe dexterity impairments had problems turning off the voice using the volume control button. The ability to change the button size and use the customized keypad was more essential for participants with severe dexterity issues.

**Table 8:** User preference for new accessibility features.

Features	Average scores				Ranking based on the average scores		
	Group 1	Group 2	Group 3	Group 1		Group 2	Group 3
Customized module list	3.7	2.2	3.2	7		1	4
Customized text display	4.0	3.8	4.4	8		7	8
Customized theme	7.3	4.5	4.2	10		10	7
Customized button size	3.6	3.0	2.8	4		4	1
Customized keyboard	3.0	3.8	3.0	2		7	2
Ability to take a picture of a pill or a bottle	4.7	3.6	3.0	9		6	2
Color-coding	3.4	3.9	3.8	3		9	6
Navigational short cuts	3.6	3.1	4.4	4		5	8
Text guidance	2.0	2.5	3.2	1		2	4
Voice guidance	3.6	2.5	5.0	4		2	10

## Discussion

### Principal Findings

The use of mHealth as a self-management intervention is a new field of research. The iMHere system is unique in that it is specifically designed to support the self-management of people with disabilities. A previous systematic review by Nussbaum et al [43] identified several mHealth apps relevant to the field of rehabilitation medicine and identified only 3 mHealth apps focused on self-management, including the iMHere system. The iMHere system has been shown to be feasible for use in the SB and SCI populations, and its use has been associated with improvements in self-management skills, caregiver assistance needed, frequency of urinary tract infections, and depressive symptoms [44,45]. In addition, Nguyen et al [46] used a web-based application to promote dyspnea self-management in persons with chronic obstructive pulmonary disease. Duggan et al [5] evaluated the SMART2 app in the self-management of chronic pain. Both mHealth apps demonstrated positive outcomes and effectiveness in self-management of the respective conditions they evaluated. However, there remains a paucity of apps focused on self-management in people with disabilities with motor, cognitive, and sensory impairments.

This study further adds to the literature on the usability of mHealth systems in people with disabilities with various dexterity-limiting disabilities, as it demonstrates that mHealth systems can be made more usable by improving efficiency, effectiveness, learnability, and user satisfaction.

Our first hypothesis addressed the usability constructs of efficiency and effectiveness (including errors). The efficiency and effectiveness of the redesigned modules were significantly better than those of the original modules, resulting in improved user performance and reduced user error. These changes were likely because of the design criteria that were implemented after careful consideration of how dexterity affects workflow and recovery from errors. The most apparent improvements in efficiency were seen in those with severe dexterity issues who benefited from text cues and color-coding of modules. These features allowed users to troubleshoot their own actions and reduce the overall error rate. Those with mild to moderate dexterity impairments benefited most from voice guidance, changes to button size, and custom keyboard options. Voice guidance, similar to text cues, also helped participants troubleshoot and reduce errors. The ability to change the target button size helped improve the user’s accuracy. The customized keyboard simplified the process of data entry. It is important to note that the improvements in efficiency gained from these features may be a result of the modules becoming more intuitive from a cognitive perspective.

Our second hypothesis addressed learnability and satisfaction. The improved usability of the redesigned modules was also evidenced by the participants’ preference for the redesigned modules. With the addition of accessibility features, we were able to further improve learnability through features such as navigational shortcuts and voice or text guidance. In addition, we added features to improve customizability, such as custom themes and lists. As seen with improvements in TUQ scores, the participants were more satisfied with the redesigned modules and would use the iMHere modules in the future. Of note, significant improvement in usability detected in the redesigned modules compared with the original modules may have been even larger because there was no ceiling effect in TUQ.

Future work on the translation of mHealth to various models of care for people with disabilities is planned. We are currently carrying out a clinical trial evaluating the community integration of people with disabilities using mHealth to supplement services provided by a community-based organization that supports independent living. We are also carrying out implementation studies to evaluate how iMHere 2.0 can be used to deliver support to caregivers of people with disabilities and those with chronic conditions and to help facilitate long-term services and support such as caregiving services.

### Study Limitations

Some limitations of this study deserve further discussion. First, we recruited a small sample, which limits the types of statistical analyses that can be performed. Second, although we redesigned all iMHere modules, this study assessed the design of only 2 modules. We chose these 2 modules because they are the most complex, containing both advanced features and basic features that are also found in the other 3 iMHere modules. As the 3 less-complex modules contain features that are similar to those tested in the more complex modules, we expect that the usability testing results for those modules would have been similar. Third, a variety of tools exist to test dexterity and usability measures. We chose the tests and measures intentionally based on the proposed usability theory but certainly, other theories, constructs, and tools are available. For instance, we did not test memorability as a measure of usability. We plan to incorporate this attribute of usability in future studies. Fourth, iMHere was not designed to support every disability or medical need, but its design is a result of research involving over 200 people with various disabilities and chronic conditions, children to older adults, and a diverse group of professionals and support personnel involved in the care of people with disabilities and chronic conditions. Finally, the participants in the study had a variety of diagnoses that resulted not only in dexterity impairments but also sensory and cognitive impairments. Thus, we were not able to determine which types of usability or accessibility issues were related to impairments other than those related to dexterity. Future studies will expand the participant population and stratify the results to further investigate the usability and accessibility needs of individuals based on their unique impairments and abilities.

### Conclusions

This study demonstrated that the iMHere mHealth system became more usable for individuals with disabilities after redesigning it according to user-centered principles. Our findings demonstrate that users became more efficient and effective when using the redesigned modules. In addition, we found that the redesigned modules were easier to use and learn for the first-time users, and users were satisfied with the redesigned modules. By including the user in the iterative process to test usability, we were able to identify features in our original module that benefited from redesign. Since the publication of this work, iMHere has launched a subsequent version (iMHere 2.0) with additional features that are focused on enhancing user experience. The associated app now integrates the family and formal caregiver interface with the client app. In addition to the existing modules, additional modules focused on physical activity, nutrition, goal setting, and education were added to the app. In the future, we hope to complete usability testing with studies that incorporate memorability into user testing. With successful implementation of iMHere among our test participants, we hope to make this app available to different disability populations in the community to promote independence of self-management with improved clinical integration to bolster continuity of care.

## Appendix

Multimedia Appendix 1 Purdue Pegboard Test results (time to complete in seconds for right, left, and both hands).

## Abbreviations

DP: difficulty-on-performance
iMHere: Interactive Mobile Health and Rehabilitation
mHealth: mobile health
PPBT: Purdue Pegboard Test
SB: spina bifida
SCI: spinal cord injury
TUQ: Telehealth Usability Questionnaire
