# Supplementary material for: User-Centered Design to Enhance mHealth Systems for Individuals With Dexterity Impairments: Accessibility and Usability Study
Source: JMIR Hum Factors. 2022 Feb 24;9(1):e23794. doi: 10.2196/23794 (PMC8914790; doi:10.2196/23794)
Supplement: Multimedia Appendix 1 [file humanfactors_v9i1e23794_app1.docx]

## Supplementary Files

Table 1. Purdue Pegboard Test results (time to complete in seconds for right, left and both hands).

| **PARTICIPANT ID** | **Right Hand** | **Left Hand** | **Both Hands** | **R+L+Both** | **Dexterity Level Group #** |
| --- | --- | --- | --- | --- | --- |
|  | Mean =17.2 | Mean =16.0 | Mean =13.4 | Mean =46.8 |  |
|  | -1SD = 15.4 | -1SD = 14.3 | -1SD = 14.3 | -1SD = 42.7 |  |
|  | -3SD = 11.8 | -3SD = 10.9 | -3SD = 10.9 | -3SD = 34.5 |  |
| P01 | 9 | 9 | 15 | 33 | 2 |
| P02 | 12 | 9 | 15 | 36 | 1 |
| P03 | 0 | 0 | 0 | 0 | 3 |
| P04 | 0 | 0 | 0 | 0 | 3 |
| P05 | 5 | 9 | 10 | 24 | 2 |
| P06 | 10 | 10 | 16 | 36 | 1 |
| P07 | 12 | 13 | 17 | 42 | 1 |
| P08 | 7 | 9 | 14 | 30 | 2 |
| P09 | 0 | 0 | 0 | 0 | 3 |
| P10 | 10 | 10 | 19 | 40 | 1 |
| P11 | 10 | 11 | 11 | 32 | 2 |
| P12 | 8 | 12 | 13 | 33 | 2 |
| P13 | 11 | 12 | 19 | 42 | 1 |
| P14 | 3 | 4 | 3 | 10 | 2 |
| P15 | 11 | 8 | 10 | 28 | 2 |
| P16 | 0 | 0 | 0 | 0 | 3 |
| P17 | 5.3 | 7 | 9 | 22 | 2 |
| P18 | 3 | 4 | 3 | 10 | 2 |
| P19 | 13 | 13 | 16 | 42 | 1 |
| P20 | 7 | 0 | 0 | 7 | 2 |
| P21 | 9 | 10 | 13 | 32 | 2 |
| P22 | 9 | 11 | 18 | 38 | 1 |
| P23 | 12 | 13 | 14 | 38 | 2 |
| P24 | 0 | 0 | 0 | 0 | 3 |
| Average (SD) | 7.4 (5.0) | 6.8 (4.4) | 9.8 (7.1) | 23.9 (16.0) |  |
